# Relative species abundance successfully predicts nestedness and interaction frequency of monthly pollination networks in an alpine meadow

**DOI:** 10.1371/journal.pone.0224316

**Published:** 2019-10-28

**Authors:** Lei Hu, Yuran Dong, Shucun Sun

**Affiliations:** 1 Department of Ecology, School of Life Sciences, Nanjing University, Nanjing, Jiangsu Province, China; 2 Center for Ecological Studies, Chengdu Institute of Biology, Chinese Academy of Sciences, Chengdu, Sichuan Province, China; University of Waikato, NEW ZEALAND

## Abstract

Plant-pollinator networks have been repeatedly reported as cumulative ones that are described with >1 years observations. However, such cumulative networks are composed of pairwise interactions recorded at different periods, and thus may not be able to reflect the reality of species interactions in nature (e.g., early-flowering plants typically do not compete for shared pollinators with late-flowering plants, but they are assumed to do so in accumulated networks). Here, we examine the monthly sampling structure of an alpine plant-pollinator bipartite network over a two-year period to determine whether relative species abundance and species traits better explain the network structure of monthly networks than yearly ones. Although community composition and species abundance varied from one month to another, the monthly networks (as well as the yearly networks described with annual pooled data) had a highly nested structure, in which specialists directly interact with generalist partners. Moreover, relative species abundance predicted the nestedness in both the monthly and yearly networks and accounted for a statistically significant percentage of the variation (i.e., 20%-44%) in the pairwise interactions of monthly networks, but not yearly networks. The combination of relative species abundance and species traits (but not species traits only) showed a similar prediction power in terms of both network nestedness and pairwise interaction frequencies. Considering the previously recognized structural pattern and associated mechanisms of plant-pollinator networks, we propose that relative species abundance may be an important factor influencing both nestedness and interaction frequency of pollination networks.

## Introduction

In theory, every species within a biological community can directly or indirectly interact with every other species to form a complex ecological network. Studies have shown that ecological networks are often endowed with distinct and repeated patterns [1, 2] that are functionally significant. For example, the structure of mutualistic networks has been demonstrated to be associated with long-term species coexistence, community diversity and stability [3, 4]. Thus, accurately identifying network pattern and exploring the mechanisms by which it forms are essential to understanding the ecological dynamics of communities [5, 6].

Plant-pollinator networks generally have a nested architecture, wherein specialist pollinators interact directly with generalist plants and *vice versa* [1]. Nested pollination networks are mostly explained by species traits (a niche process) and relative abundance (a neutral process), as noted in a number of studies [7–9]. Species trait- matches or mismatches have been hypothesized and demonstrated to be the mechanism by which pairwise interactions can or cannot arise [9, 10]. Because variation in species composition is not necessarily consistent between plants and pollinators (e.g., plant species composition may remain similar while pollinator species composition may change dramatically between or among successive years [11–13]), relative species abundance has been also frequently suggested to be responsible for the nested structure of a network [11, 13] and even responsible for the species-pairwise relationships of pollination networks [8, 14]. Nevertheless, despite the success of predicting the nested architecture of pollination networks [13, 15–17], relative species abundance explains very little of the frequency of species pairwise interactions in pollination networks [18–21]. Even in the models incorporating phenology and species traits, it cannot successfully predict the interaction frequencies of pollination networks [8, 9, 19].

One of the potential causes responsible for the unsuccessful prediction of interaction frequencies is that pollination networks are often described with data accumulated over several consecutive years (because flowering plants and pollinators are highly dynamic with large temporal variation in species composition and abundance). For example, Price et al. (2005) surveyed flower visitors over seven summers in a montane habitat [18]; Fang and Huang (2012) examined pollination networks over four consecutive years in an alpine meadow [21]; Chacoff et al. (2018) investigated plant-pollinator networks over six consecutive years in a Monte Desert ecoregion [14]. Unfortunately, using pooled data may not be able to reflect the reality of species interactions [22]. For example, early-flowering plants do not necessarily compete for shared pollinator species with late-flowering plant species if their flowering phenologies do not overlap [23]. Such a plant-plant competition (for shared pollinators) within cumulative pollination networks does not exist in biological reality, and instead they may positively interact, e.g., an early flowering species can provide resources for multivoltine pollinators that pollinate late-flowering species. Importantly, such an error may raise problems in models incorporating phenology as long as accumulative networks are used. For example, if early-season plant species are much more abundant than the late-season ones, models would predict an extremely low frequency for the interaction between the late-season species and their shared pollinators. This prediction could be wrong if the shared pollinators are more abundant in late seasons and hence the late-season plant species could be more frequently pollinated. Thus, networks described with short term data should be examined to determine whether relative species abundance may account for nestedness and pairwise interactions in pollination networks [24].

Here, we investigated the plant and pollinator assemblages and their pairwise monthly interactions during two growing seasons in a Tibetan alpine meadow. The primary objective of this study is to determine whether relative species abundance together with species traits can better predict the structure of monthly pollination networks than yearly ones. Specifically, we determined whether these networks were consistently nested for both monthly and yearly networks and tested whether relative species abundance and species traits could predict the nestedness and pairwise interactions of pollination networks. We also calculated the frequency of pairwise interactions for both monthly and yearly networks and asked whether relative species abundance and species traits significantly accounted for the variation in the interaction frequency. As noted, we hypothesized that relative species abundance and species traits would be better predictors for the interaction frequency of monthly networks than yearly ones.

## Materials and methods

### Study site

The study site was conducted in an alpine meadow in Hongyuan County (N 32°48', E 102°33'; altitude ≈ 3500 m), Sichuan Province, China, in the eastern part of the Tibet Plateau. The study site has a typical continental plateau climate, with a mean annual temperature of 0.9°C, mean annual precipitation of 744 mm, which mostly occurs during growing seasons from late May to September. The soil is often high in organic content (250 g·kg^-1^), while low in total nitrogen and phosphorus, with 8 g·kg^-1^ and 5 mg·kg^-1^, respectively [25].

The study site is mostly dominated by sedges including *Kobresia setchwanensis* and Asteracea species (e.g. *Saussurea nigrescens*, *Anaphalis lacteal*, *Taraxacum* sp1). Asteraceae species are particularly diverse (>30 species) and abundant. The average vegetation coverage is over 90%, and the average plant height is about 30 cm. Previous studies have shown that the alpine meadow is taxonomically diverse in insect herbivores and pollinators such as bumblebees, flies, hoverflies, moth and butterflies [26].

### Pollination network

The investigation of pollination network was conducted in an alpine meadow (200 m× 200 m in area) during the growing season from June to September of 2016 and 2017. Flower-visiting insects were observed and recorded for over two hours along a pair of 200 m transects, sixteen transects per day. A plant-pollinator interaction was recorded only if an insect contacts the reproductive structure of a plant and actively searched for pollen and/or nectar [9, 27]. Field work was carried out in clear days from 9:00–17:00, during which insects had extremely high visitation activities at suitable air temperatures.

Many pollinators are hard to identify and some of them have never been recorded before. We collected pollinator specimens and identified them by DNA barcoding technology. DNA extraction was conducted using a commercial kit (TIANcombi DNA Lyse&Det PCR Kit). COI sequences were obtained using the universal barcoding primers LCO1-490 (5’-GGTCAACAAATCATAAAGATATTGG-3’) and HCO2-198 (5’-TAAACTTCAGGGTGACCAAAAAATCA-3’) [28]. The total volume of the PCR amplification system was 20 μl, including 10 μl I-5TM High-Fidelity Master Mix (MCLAB, San Francisco, U.S.A.), 1 μl primer, 1 μl DNA template, and 8 μl ddH2O. The reaction conditions were 98°C for 2 min, 98°C for 10 s, 45°C for 10 s, and 72°C for 20 s for 40 cycles, followed by 72°C for 5 min. PCR products were detected on a 1% agarose gel, run on an ABI 3730 xl DNA Sequencer.

The sequences were checked and edited in Sequencher version 4.1.4 (Gene Codes Corporation, Ann Arbor, Michigan, USA), and aligned using Geneious Pro version 4.8 (Biomatters Ltd., Auckland, New Zealand). A Neighbor-Joining (NJ) tree based on a K2P distance model was generated to provide a clustering pattern of our sampled pollinator in MEGA V. 7.0 [29]. Sequences were then compared with GenBank nucleotide database using BLAST (http://www.blast.ncbi.nlm.nih.gov/Blast.cgi).

### Relative species abundance

We independently estimated floral density per m^2^ for plant species every five days in more than fifteen 1-m^2^ quadrats placed randomly along the transects. Two techniques were used to estimate pollinator species abundance: sweep-netting and pan-trapping [30, 31]. A 2 meters long sweeping-net with a 37 cm net diameter was used to collect foraging pollinators along the transects for 5 min with about 100 sweeps, during which we maintained a constant pace and frequency. Pan-trapping was conducted using 10 plastic pans (22 cm diameter, 3 cm height, and 750 ml filled with about 250 ml of soapy water) placed 50 m apart along transects within the sampling plot. The pans were placed out from 9:00 AM to 18:00 PM. Sweep-netting and pan-trap sampling was carried out for 16 transects (each was 200m long and 2m wide) every five days.

### Species traits

We measured plant flower depth for all the following plant species and leg length for all the pollinator species because flower depth and pollinator leg length are crucial to the formation of plant-pollinator pairwise interaction [9]. All the flowering plant species, based on the flower depth, were assigned to one of four size classes (i.e. disk florets, small, medium, and large corolla/nectar spur). All the pollinator species, based on the leg length, were also assigned to one of four size classes (i.e. minute, short, medium, and long). The four classes were 1) < 2 cm, 2) 2–4 cm, 3) 4–9 cm, and 4) >9 cm for both flower depth and pollinator leg length. Flower depth and pollinator leg length are measured using calipers with a representative sample (> 10 individuals) for each plant and pollinator species, respectively. When the class of pollinator leg length was equal or higher than the class of flower depth, the two species were considered interreacting with each other because the legs of pollinators were often placed inside flower corolla tubes [9]. Species traits measurements were conducted in 2017 only.

### Data analysis

We analyzed data at both the monthly and yearly scales. In the alpine meadow, almost all of the plant species started their flowering season from the beginning of June or the end of May, and moreover, the flowering period of most species ranged between 25 and 35 days. For example, the dominant species such as *Potentilla anserina* (mainly flowered in June), *Anemone rivularis* (mainly in July), *Saussurea nigrescens* (mainly in August), and *Allium sikkimense* (mainly in September) had a flowering period of 25, 31, 35 and 34 days, respectively. Accordingly, we choose the monthly scale to study network structure.

To visualize dynamic changes in plant, pollinator community composition, pairwise interactions, and plant and pollinator species traits each month, we conducted multidimensional scaling (NMDS) based on the Bray-Curtis dissimilarity index (metaMDS function, “Vegan” package for R). Stress values below 0.2 indicated that the ordination adequately represented the data [32]. A measure of goodness of fit based on the Bray-Curtis dissimilarity index and distance in graphical representation was selected to evaluate the NMDS results, where the smaller the circle, the better goodness of fit [33].

Using plant-pollinator visitation frequencies, a quantitative plant-pollinator interaction matrix *Y* = [*y*_*ij*_] at a monthly scale was constructed, with rows corresponding to plant species and columns corresponding to pollinator species; cell entries *y*_*ij*_ were integers representing the number of flowers in plant species *i* visited by pollinator species *j*. Then, the parameters “nestedness” and “weighted NODF”, which describe the extent to which more specialized species interacted with a proper subset of species generalists [34, 35], were calculated for each monthly pollination matrix using the bipartite R statistical software package. Both “nestedness” and “weighted NODF” varied from 0 to 100. “nestedness” used qualitative data, with 0 indicating perfect nestedness and 100 indicating randomly distributed interactions, whereas “weighted NODF” used quantitative data incorporating interaction frequencies, with 100 indicating perfect nestedness and 0 indicating randomly distributed interactions. Finally, null models were used to test the significance of the nested structure [36]. We first constructed 1000 simulated networks using the “shuffle.web” function in R “bipartite” package [37], and then we calculated the confidence intervals of “nestedness” and “weighted NODF” for the simulated networks [34]. When the “nestedness” of the observed matrix was lower than the minimum of the confidence intervals, or the “weighted NODF” was higher than the maximum, the nested structure of observed network was significant [8]. We did not calculate other network parameters (e.g. connectance, interaction evenness, linkage density) because this study was designed to provide a comparison with previous studies that have reported that relative species abundance was a good predictor for nestedness but not the interaction frequencies of accumulated pollination networks.

In order to test whether the relative species abundance and species traits fit the observed network structure parameters, we first constructed a probability matrix *X* based on the relative species abundance or species trait matches, where entries *x*_*ij*_ were the probability of occurrence for each pairwise interaction. For the matrix based on relative species abundance *x*_*ij*_ was simply the product of the relative abundance of a plant species *i* and a pollinator species *j*, while in the matrix based on species traits *x*_*ij*_ was set to be 1 or 0, indicating the match or mismatch between the species traits of plants and pollinators, respectively. The matrix combined species traits and relative species abundance were formulated as the element-wise product of the two single-factor matrices. Because the predicted matrix based on relative species abundance was not bounded between 0 and 1, we used the function ‘mgen’ the R “bipartite” package generate simulated matrices [8]. The “nestedness” and “weighted NODF” of the predicted and observed matrices were calculated, respectively. Their significance was tested using null models as aforementioned. The predicted “Nestedness” and “weighted NODF” was supposed to be indistinguishable from the observed ones if they were both significant or non-significant.

In order to test the contribution of relative species abundance and species traits on the interaction frequency, the observed and predicted matrices were compared using function ‘dmultinom’ in the stats package of R language and then Akaike information criterion (AIC) was calculated [8]. △AIC, which was the AIC for probability matrix 1 minus the AIC for the best fitting probability matrix (which by definition has △AIC = 0), was used to verify the goodness of fit for the matrices predicted based on species traits, relative species abundance and their combination. In addition, we constructed 10 000 probability matrices by assigning a random probability (between 0 and 1) to each *a*_*ij*_ element using the “*runif*” function of R software, and then calculated AIC values for each random matrix. If a AIC value was greater than the lower interval of a 95% confidence interval of 10 000 matrices, its model was thought not significantly contribute to explaining the observed interaction frequencies [38]. The general linear regression between observed and predicted interaction frequency of the matrix with the lowest AIC were analyzed to obtain the relative contribution on pairwise interactions of nested pollination networks [9].

## Results

### Dynamic pollination network composition

**Fig 1:**
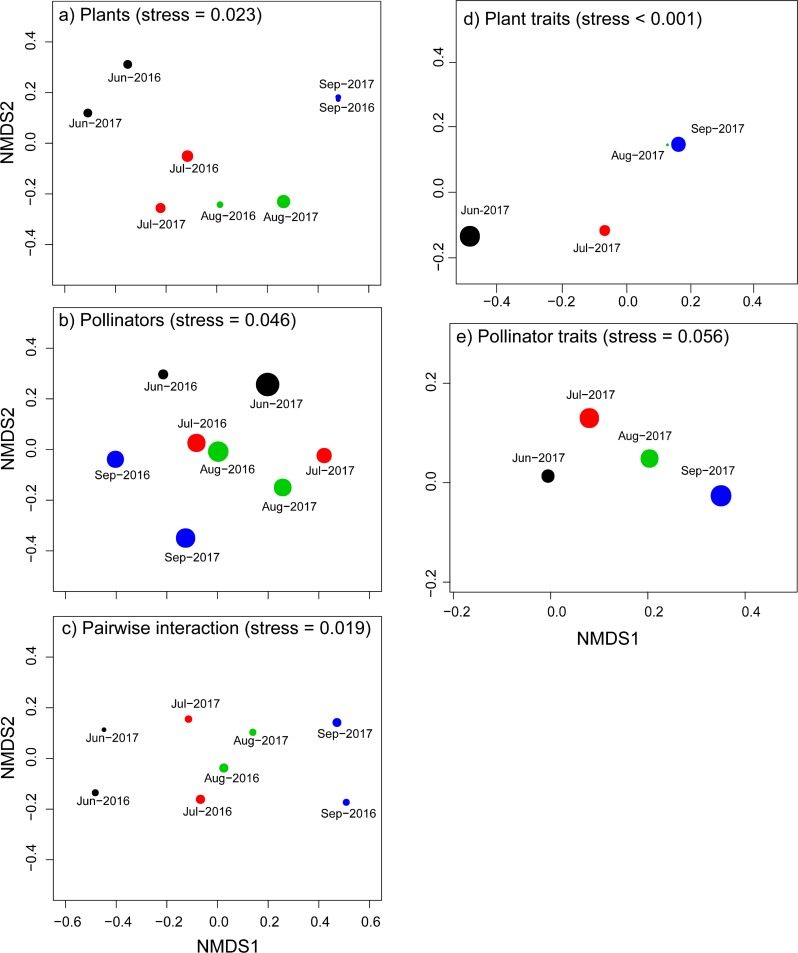
**Dynamic changes in community composition and species traits, as shown by nonmetric multidimensional scaling (NMDS) of plant (a), pollinator (b) community composition, pairwise interactions (c), plant traits (d), and pollinator traits (e) in each month.** Different colors indicated the different months. The closer the two points are, the more similar they are. The smaller the points are, the better the goodness of fit are.

Plant species composition differed among months in both 2016 and 2017, whereas the plant species of each month were generally similar between the two years (Fig 1A). Moreover, the difference was also strong between the observation years (Fig 1B). Interestingly, the pollinator species composition in June was fairly similar to July and September in both 2016 and 2017 (Fig 1B), possibly because some of the pollinator species were multivoltine. In addition, a strong difference of pairwise interactions was observed between months and between years (Fig 1C). Plant traits differed largely among June, July and August of 2017 (Fig 1D), whereas the monthly difference in pollinator traits was smaller albeit marginally significant (Fig 1E).

### Effects of relative species abundance and species traits on network nestedness

**Fig 2:**
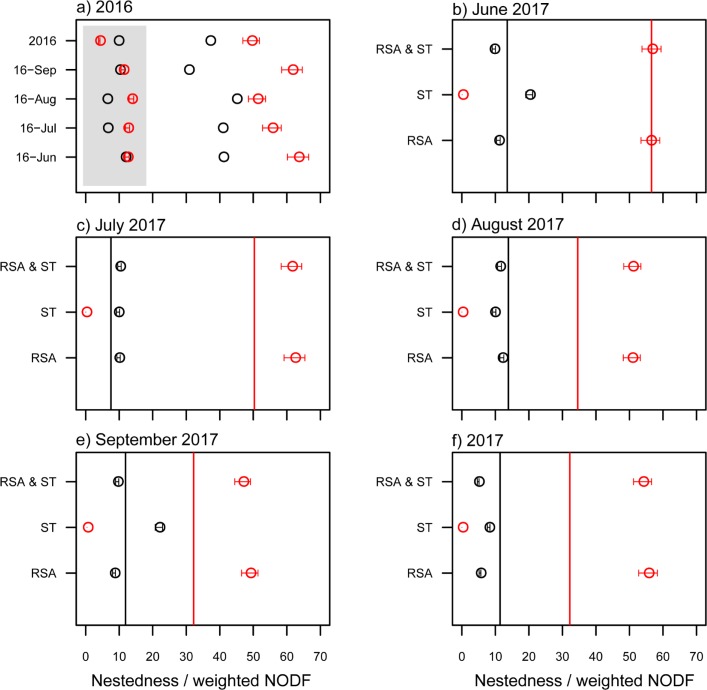
Comparison between observed and predicted structure parameters (estimate ± 95% confidence intervals) for the monthly and yearly networks. RSA, ST, and RSA & ST indicate the predicted pollination network based on the relative species abundance, species traits, and both, respectively. Nevertheless, the predicted network was based on RSA only in 2016 (a), where the black and red circles indicate the observed and predicted values, respectively, and the circles in the shaded and light areas are for “nestedness” and “weighted NODF”, respectively. For the other panels of 2017, the black and red circles, as well as the black and red lines, indicate the predicted “nestedness” and “weighted NODF”, respectively.

The observed pollination network was significantly nested in each month and each year, as indicated by both of the parameters “nestedness” and “weighted NODF” (Table 1). The structure of the pollination networks predicted by relative species abundance were also highly nested for each month and each year, despite the predicted “weighted NODF” being higher than that observed (Fig 2A–2D). In the monthly network of 2017, the nested structure of qualitative network (‘nestedness’) was also predicted by species traits and the combination of relative species abundance and species traits, respectively (Table 1, Fig 2E and 2G). Moreover, the nested structure of quantitative network (‘weighted NODF’) was predicted by the combination of relative species abundance and species traits (Table 1 and Fig 2F) but not species traits (Table 1 and Fig 2H).

**Table 1:** The dynamic pollination network metrics (Nestedness and Weighted NODF) of both observed networks and predicted networks based on relative species abundance in each month and each year of 2016 and 2017.

			Nestedness		Weighted NODF	
			Observed	Predicted	Observed	Predicted
2016	RSA	June	11.69(40.19–60.37)	12.35(50.81–70.64)	40.87(6.00–12.16)	63.35(13.76–20.33)
		July	13.57(52.78–69.23)	12.47(63.68–76.25)	34.09(8.50–11.56)	55.54(16.45–19.84)
		August	6.37(45.28–59.98)	13.67(63.75–77.14)	40.68(6.08–8.76)	51.1(11.86–14.64)
		September	7.49(46.06–64.55)	8.38(56.62–71.77)	50.31(7.30–11.10)	61.53(18.02–22.32)
		Year	9.61(56.42–67.69)	4.06(56.81–67.87)	36.95(7.98–9.65)	49.35(7.05–8.53)
2017	RSA	June	6.24(46.20–59.77)	10.21(65.37–77.58)	44.86(6.74–9.05)	56.24(13.05–15.89)
		July	13.49(61.74–73.80)	9.81(71.25–80.13)	34.57(9.59–11.45)	62.27(16.48–18.65)
		August	10.04(46.98–56.99)	11.91(66.42–76.88)	30.57(6.15–7.71)	50.69(9.81–11.61)
		September	11.67(42.08–58.22)	10.89(60.55–74.08)	32.2(6.32–9.78)	48.96(10.32–14.11)
		Year	13.7(62.39–71.21)	5.42(69.00–77.20)	30.82(8.58–9.74)	55.52(9.52–10.65)
	Species traits	June		14.26 (17.06–23.68)		0.09 (0–0.25)
		July		9.64 (13.75–18.42)		0.03 (0–0.15)
		August		9.67 (12.18–16.19)		0.02 (0–0.03)
		September		21.82 (23.30–34.95)		0.40 (0–0.48)
		Year		7.99 (9.82–12.52)		0.03 (0–0.08)
	RSA & Species traits	June		9.52 (65.28–77.40)		56.58 (13.63–16.53)
		July		10.08 (71.24–80.21)		61.37 (15.90–18.04)
		August		11.23 (65.93–76.55)		50.85 (9.98–11.77)
		September		9.39 (54.41–70.94)		46.82 (9.46–13.37)
		Year		4.87 (67.25–76.09)		53.92 (8.99–10.17)

Comparisons of observed vs. predicted indicate that relative species abundance is a good indicator for network nestedness. The value ranges within the parentheses are the 95% confidence intervals of null modes based on the observed and predicted network.

### Effects of relative species abundance and species traits on pairwise interaction

The effect of relative species abundance on pairwise interaction frequencies was significant and more pronounced than that of species traits for each month in both years, but not for the whole growing season in either year. (Table 2). The effect of the combination of species traits and relative species abundance was comparable to that of relative species abundance (Table 2).

**Table 2:** Results of likelihood analyses showing whether relative species abundance (RSA), species traits, or their combination (Species traits & RSA), is a good indicator for pairwise interaction.

		Model	Likelihood	AIC	CI	△AIC
2016	June	RSA	1261.22	2524.44*	5644.08–9304.94	
	July	RSA	3574.61	7151.21*	7518.00–9923.86	
	August	RSA	3746.635	7495.27*	14371.35–20038.36	
	September	RSA	2075.37	4152.75*	8459.42–11204.42	
	Year	RSA	62474.66	124951.3	37948.62–46703.28	
2017	June	RSA	5888.5	11778.99*	23657.34–26579.30	0
		Species traits & RSA	5981.98	11965.96*	17271.07–21329.57	186.97
		Species traits	12259.03	24520.07	2481.96–2627.26	12554.11
	July	RSA	16272.93	32547.85*	32850.97–39894.87	0
		Species traits	16461.31	32924.62	4114.69–4300.91	376.77
		Species traits & RSA	16587.4	33176.8	23505.05–27220.19	252.18
	August	RSA	8939.22	17880.45*	31373.03–40668.37	0
		Species traits & RSA	9716.98	19435.95*	24354.33–29407.44	1555.5
		Species traits	16593.92	33189.83	4581.43–4679.59	13753.88
	September	RSA	2565.42	5132.83*	7973.96–10495.05	0
		Species traits & RSA	7765.81	15533.16	6357.04–7173.74	10400.33
		Species traits	9329.37	18660.74	1349.58–1462.61	3127.58
	Year	RSA	76864.04	153730.1*	98486.84–116101.3	0
		Species traits & RSA	83038.6	166079.2	95176.09–110788.30	12349.1
		Species traits	315285.9	630573.7	206985.2–306478.3	464494.5

The AIC values designed with * denote that relative species abundance significantly contributed to the variation in pairwise interaction. CI indicates the 95% confidence intervals of AIC of 10 000 random probability matrices.

**Fig 3:**
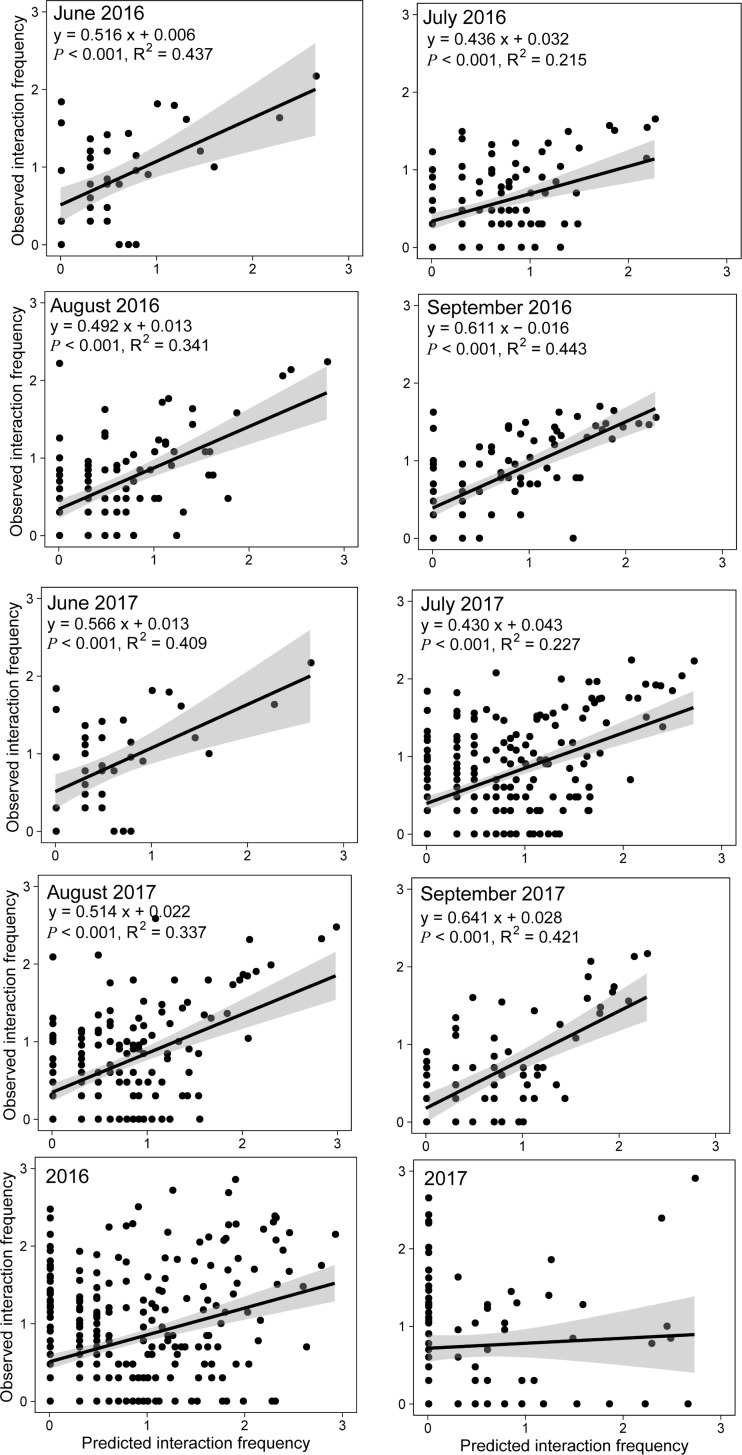
The linear regression between observed and predicted interaction frequency based on the relative species abundance. The solid lines show the fit of a quadratic model and the 95% confidence interval.

Nevertheless, the matrix based both relative species only was the most appropriate one (with the lowest AIC) predicting pairwise interaction frequencies. Relative species abundance accounted for 20%-44% of the variation in the interaction frequency in both years (Fig 3). Moreover, relative species abundance accounted for more of the variance in the interaction frequency in the early and in the late season (June and September) than the middle (July and August).

## Discussion

Our data show that community species composition consistently changes during the growing season, indicating that pairwise interactions and associated pollination networks varied among months and between years. However, the pollination network manifested a nested structure in each month and each year. Moreover, consistent with our hypothesis, relative species abundance (RSA), as well as the combination of RSA and species traits, was a good predictor of network nestedness. In addition, RSA partly but significantly explained the interaction frequency of monthly networks (albeit not for the whole growing season), with a higher explanation efficiency than the combination of RSA and species traits.

The constant nestedness of monthly and yearly (accumulative) pollination networks is consistent with a number of previous studies addressing mutualistic networks [1, 14, 21, 39] showing that nestedness is a structural property regardless of species composition and the configuration of pairwise interactions. It is possible that many configurations of pairwise interactions may give the same value of ‘nestedness’ and ‘weighted NODF’ in the network metrics. Some studies have successfully predicted nestedness but not pairwise interactions [8–9, 40]. The success of RSA predicting nestedness may be attributable to the fact that the effect of RSA overrides that of species composition. We have observed that plant species differ significantly from month to month, whereas pollinator species are similar between months, particularly in July and August. Consistently, the monthly difference is greater in plant traits than pollinator traits. Yet, networks manifest highly nested structures in all months. The mechanism underlying the effect of RSA on nestedness could be simply because of the right-skewed frequency distribution of relative species abundance of both plants and pollinators (S1 Table), which should lead to nested networks according to the neutral process hypothesis [12, 13, 15, 23, 24]. Nevertheless, the RSA alone overpredicted nestedness for all the monthly and yearly networks, as indicated by Canard et al. (2014) who showed that RSA overestimated nestedness and failed to predict the natural variation of nestedness values [15]. Indeed, the combination of RSA and species traits also predicted the network ‘nestedness’ and ‘weighted NODF’ than species traits. Moreover, species traits alone significantly explained the qualitative network ‘nestedness’, further indicating the contribution of species traits to network structural properties. RSA significantly explained part of the variation in the interaction frequency of monthly networks, but not the yearly (accumulative) networks. This is presumably because the monthly networks reflected the biological reality more than the yearly ones. Monthly networks describe short-period interactions, within which plants and pollinators are active at the same time and hence likely to create strong pairwise interactions, reflecting biological reality in nature. In contrast, yearly networks are described using accumulated data including the plants and pollinators that might not be active at the same time and thus often fail to correctly predict the frequency of pair-wise interactions (see Introduction). Incorrect estimates can be large because some pollinators (e.g., bumblebees, *Apis mellifera*, and *Peleteria iterans*) are often active and can pollinate plants in different months. The problem can negatively affect the relationship between plants and pollinators in the yearly networks. As a test, we calculated the RSA of the pollinators based on the sum of observations and determined the predictive power of RSA regarding interaction frequency. We found that this RSA could not significantly predict the frequency of pairwise interactions for the yearly networks (Fig 3). Indeed, modelling has demonstrated that RSA is a poor indicator for the interaction frequency of cumulative pollination networks, although it can predict network nestedness [9, 38, 41, 42]. It is worth noting that RSA explained only a small part of variation in the interaction frequency of the monthly networks. This is probably because including RSA only (while neglecting niche process) overpredicts the opportunity of forming a pairwise interaction [43].

It is worthwhile to note that species traits showed little power to predict the pairwise interaction frequencies. This is perhaps because biological constraints (species trait mismatch) are not crucial to the formation of most pairwise interactions (except for few; e.g. *Bombus friseanus* primarily visiting *Pedicularis* spp.). For example, most of the plant species belonging to Compositae and Ranunculaceae are not highly specialized to particular pollinator species but are able to be visited by many different pollinator species with varying leg lengths. To this end, the species trait effect could be overridden by the RSA effect on pairwise interaction frequencies, as indicated by the fact that the RSA effect alone was comparable with the combination of RSA and species traits and much greater than the species trait effect that proved non-significant. Nevertheless, species traits could be more powerful for the prediction of the network structure, if important traits like pollen presentation pattern, flower dimensionality, accessibility of nectar, and flower orientation [16] had been included in this study.

In summary, we have demonstrated that both monthly and yearly networks have nested structures. Incorporating this finding in those of numerous previous network studies indicates that nestedness is widespread in pollination networks. Moreover, our data clearly show that RSA but not species traits can significantly account for the variation in the interaction frequency of monthly networks and is more accurate in predicting interaction frequency in comparison to yearly networks. Thus, describing and evaluating short-term networks may be preferable in further network analyses. Additionally, the importance of RSA to network structure as revealed in this study motivates us to speculate that RSA may not simply affect the frequency by which specific plant-insect interact, but also possibly serves as a selective force for both plants and pollinators to form pairwise interactions if their abundance is evolutionarily stable.

## Supporting information

S1 Table The skewness of frequency distribution of relative species abundance in both plant and pollinator communities. (DOCX)
