# Supplementary material for: Relative species abundance successfully predicts nestedness and interaction frequency of monthly pollination networks in an alpine meadow
Source: PLoS One. 2019 Oct 28;14(10):e0224316. doi: 10.1371/journal.pone.0224316 (PMC6816544; doi:10.1371/journal.pone.0224316)
Supplement: S1 Table — (DOCX) [file pone.0224316.s001.docx]

**S1 Table. The skewness of frequency distribution of relative species abundance in both plant and pollinator communities.**

|  | | Plant abundance distribution | | Pollinator abundance distribution | |
| --- | --- | --- | --- | --- | --- |
|  |  | Skewness | *P* | Skewness | *P* |
| 2016 | June | 2.19 | <0.001 | 2.30 | <0.001 |
|  | July | 2.25 | <0.001 | 2.97 | <0.001 |
|  | August | 2.50 | <0.001 | 3.55 | <0.001 |
|  | September | 1.29 | 0.013 | 0.91 | 0.035 |
|  | Growing seasons | 2.38 | <0.001 | 4.30 | <0.001 |
| 2017 | June | 2.25 | <0.001 | 2.97 | <0.001 |
|  | July | 1.40 | 0.001 | 3.69 | <0.001 |
|  | August | 3.08 | <0.001 | 4.62 | <0.001 |
|  | September | 2.62 | <0.001 | 2.68 | <0.001 |
|  | Growing seasons | 2.66 | <0.001 | 6.63 | <0.001 |
